# Multibacillary leprosy by population groups in Brazil: Lessons from an observational study

**DOI:** 10.1371/journal.pntd.0005364

**Published:** 2017-02-13

**Authors:** Mauricio Lisboa Nobre, Ximena Illarramendi, Kathryn Margaret Dupnik, Mariana de Andrea Hacker, José Augusto da Costa Nery, Selma Maria Bezerra Jerônimo, Euzenir Nunes Sarno

**Affiliations:** 1 Pós-Graduação em Medicina Tropical, Instituto Oswaldo Cruz, Fiocruz, Rio de Janeiro, RJ, Brazil; 2 Hospital Giselda Trigueiro, Secretaria Estadual de Saúde, Natal, RN, Brazil; 3 Instituto de Medicina Tropical do Rio Grande do Norte, Universidade Federal do Rio Grande do Norte, Natal, RN, Brazil; 4 Centro de Desenvolvimento Tecnológico em Saúde, Fiocruz, Rio de Janeiro, RJ, Brazil; 5 Division of Infectious Diseases and Center for Global Health, Weill Cornell Medical College, New York, NY, United States of America; 6 Laboratório de Hanseníase, Instituto Oswaldo Cruz, Fiocruz, Rio de Janeiro, RJ, Brazil; 7 Departamento de Bioquímica, Centro de Biociências, Universidade Federal do Rio Grande do Norte, Natal, RN, Brazil; 8 Instituto Nacional de Ciência e Tecnologia de Doenças Tropicais, INCT-DT, Natal, RN, Brazil; Fondation Raoul Follereau, FRANCE

## Abstract

**Background:**

Leprosy remains an important public health problem in Brazil where 28,761 new cases were diagnosed in 2015, the second highest number of new cases detected globally. The disease is caused by *Mycobacterium leprae*, a pathogen spread by patients with multibacillary (MB) leprosy. This study was designed to identify population groups most at risk for MB disease in Brazil, contributing to new ideas for early diagnosis and leprosy control.

**Methods:**

A national databank of cases reported in Brazil (2001–2013) was used to evaluate epidemiological characteristics of MB leprosy. Additionally, the databank of a leprosy reference center was used to determine factors associated with higher bacillary loads.

**Results:**

A total of 541,090 cases were analyzed. New case detection rates (NCDRs) increased with age, especially for men with MB leprosy, reaching 44.8 new cases/100,000 population in 65–69 year olds. Males and subjects older than 59 years had twice the odds of MB leprosy than females and younger cases (OR = 2.36, CI95% = 2.33–2.38; OR = 1.99, CI95% = 1.96–2.02, respectively). Bacillary load was higher in male and in patients aged 20–39 and 40–59 years compared to females and other age groups. From 2003 to 2013, there was a progressive reduction in annual NCDRs and an increase in the percentage of MB cases and of elderly patients in Brazil. These data suggest reduction of leprosy transmission in the country.

**Conclusion:**

Public health policies for leprosy control in endemic areas in Brazil should include activities especially addressed to men and to the elderly in order to further reduce *M*. *leprae* transmission.

## Author summary

Leprosy is caused by *Mycobacterium leprae*, a bacillus transmitted by patients with multibacillary (MB) disease. Men and elderly are more likely to have MB leprosy, which has been attributed to an increased exposure to infection by male sex, decreased access of men to healthcare resulting in delayed diagnosis and long incubation period for MB disease. In this study, we found that the odds ratio for MB leprosy is two-fold higher for men than for women in all Brazilian states, independent of their endemic level. The same was observed for patients aged 60 or older compared to younger cases. Detection rates for MB leprosy remained higher for men and elderly patients timely detected (without physical disabilities), showing that late diagnosis is not enough to explain this association. Additionally, we showed that *M*. *leprae* load is higher in men than in women, despite early detection. These findings are relevant, because main activities to diagnose new cases of leprosy in Brazil have been focused on school surveys, detecting children who most likely have paucibacillary leprosy, which is non-contagious. To prevent transmission within the community, additional activities need to include groups at greater risk for MB leprosy. As such, we suggest that specific strategies for disease control should be adopted to effectively reach males and the elderly in endemic areas for leprosy.

## Introduction

Leprosy is a chronic disease caused by *Mycobacterium leprae* infection [1]. In 2015, 210,758 new cases of leprosy were diagnosed in 136 countries and territories worldwide, of which 8.9% were in children [2]. Between 2007 and 2015, more than 110,000 people diagnosed with leprosy had physical deformities at diagnosis, an indicator of delayed detection [2,3].

The global number of leprosy new cases sharply dropped by 75% during the period of 2000–2006, which reflects especially an abrupt fall of cases detected in India, a country that contributes more than 50% of the world new cases annually [2,3]. Some authors have attributed this decrease to a reduction in active case finding activities and the adoption of new strategies to effectively reduce transmission of *M*. *leprae* has been emphatically recommended [4,5].

Leprosy presents a wide range of clinical manifestations, but for treatment purposes a simplified field operational classification based on the number of skin lesions is available: paucibacillary (PB) leprosy has one to five skin lesions, and multibacillary (MB) patients have six or more skin lesions [6]. Bacillary index based on the slit-skin smear analysis is a more accurate means of classification, but usually it is available only in more specialized health centers [6,7].

MB leprosy occurs in people with weak cell-mediated immune response against *M*. *leprae*, who develop high bacillary load and become the main source of infection [8]. Therefore, strategies to stop transmission should include measures to diagnose and treat MB cases. However, screening campaigns generally search for skin lesions with loss of sensation, even though in 30% of patients, especially in those with MB leprosy, this finding can be absent [9]. Surveys in schools are used as a strategy for early diagnosis [10], but MB cases are less common in children [11]. Thus, a question remains: Are current control strategies successful in effectively stopping *M*. *leprae* transmission?

In 2015 Brazil detected 28,761 new cases of leprosy, the second highest number of cases in the world [2,12]. The disease is still highly endemic in different areas of the country where its control must be improved. For these reasons, this study aimed to analyze epidemiologic patterns of MB cases in Brazil, in order to gather data to develop additional strategies to decrease *M*. *leprae* transmission.

## Methods

### Study design

This is an observational analytic non-concurrent secondary data study. Data of the national government database of leprosy cases diagnosed in Brazil (2001–2013) were used for the epidemiological trend evaluation of MB cases. The database is generated in all Brazilian States that have diverse capacities and expertise for leprosy diagnosis and classification. Therefore, to have a homogeneous group classified under the same standards, we included an analysis of bacillary loads obtained from a database at a leprosy referral center. At Souza Araújo Outpatient Clinic (Ambulatório Souza Araújo, ASA/Fiocruz, Rio de Janeiro, Brazil), slit skin smears are collected and read under standard protocols and only cases with positive bacilloscopy are classified as multibacillary. For this analysis we chose a different period of time (1990 to 2014) to include the largest possible number of cases.

### Source of data

Brazil monitors mandatory notifiable conditions using SINAN (National Notifiable Diseases Information System). For each new case, a report form is generated at local health units, and sent to the central health services where data are checked, digitized and transferred from municipalities to States, and to the national database [13]. Until mid-2015 de-identified information in the national databases was freely accessible through the Informatics Department of the Public Health Care System (DATASUS).

All data were obtained from DATASUS on June 2, 2015. New cases, defined by the Ministry of Health as cases of leprosy with no previous treatment [7], were included. Sex, age group and operational classification were used as classifying variables. Cases with missing information regarding one of these variables were excluded. Physical disabilities caused by leprosy were classified as grade zero when patients had no problem in eyes, hands or feet; grade one when there was anesthesia in hands, feet or eyes; and grade two when there were visible deformities in hands, feet or eyes, or severe visual impairment [14]. As disability grade is an indirect indicator of diagnosis delay [9], rates were calculated independently for patients without physical impairment (disability grade 0) to reduce the influence of late diagnosis in progression to MB leprosy. Given that detection rates for MB leprosy have been related to leprosy endemicity, which is variable in Brazil, we looked for the relation of sex, age groups and MB rates by state.

To evaluate bacillary load variations, all new cases treated at ASA/Fiocruz (1990 to 2014) were analyzed using de-identified data on sex, age, disability grade and bacillary index (BI) at diagnosis. BI is calculated using Ridley’s logarithmic scale for number of acid-fast bacilli per microscopic field in smears collected from ear lobes, elbows, and leprosy skin lesions. In this scale, each additional unit indicates a 10-fold increase in the number of bacilli and is scored from 0 to 6+, ranging from no bacilli per 100 microscopic fields to 1,000 or more bacilli per microscopic field. Final BI results are calculated as the arithmetic mean of skin sites collected in each patient [15]. In ASA/Fiocruz, four or six sites are examined, generally two ear lobes, one elbow and a cutaneous lesion. Cases classified as indeterminate or pure neural leprosy, which present higher probabilities for misdiagnosis, and those with missing information regarding any variable were excluded.

### Calculation of epidemiological indicators

National data on leprosy were categorized by year of diagnosis, sex and age group. Population data were obtained from the IBGE (Brazilian Institute of Geography and Statistics) [16]. Age was assigned with DATASUS age group definitions: 0–4, 5–9, 10–14, 15–19, 20–39, 40–59, 60–64, 65–69, 70–79, and 80 or more years of age.

New case detection rate (NCDR) is the number of new cases per 100,000 people, per year. Sex ratio is the quotient of NCDR in men to NCDR in women. Mean NCDRs were calculated as the arithmetic mean of annual new cases from 2001–2013, divided by the population in mid-2007. Mean NCDRs were calculated by sex and by age group using arithmetic mean of cases (2001–2013) and corresponding population at mid-2007. Mean NCDRs were also calculated by operational classification.

Mean NCDRs by age groups were calculated separately for three time periods: 2002–2005; 2006–2009 and 2010–2013, to ascertain whether there were changes in age distribution related to reduction in NCDRs.

### Statistical analysis

Data were exported to Microsoft Excel worksheets (version 14.0.4760.1.000/2010). Statistical analysis was performed in Openepi (version 3.03a, CDC, Atlanta, Georgia, USA, available at http://www.openepi.com) and SPSS (version 22, IBM Corp, Armonk, NY, USA) using chi (χ)^2^ test (Pearson) to compare rates between selected groups. Annual coefficient means were compared between men and women for all age groups using analyses of variance (ANOVA). Linear regression was used to test gender differences for the NCDR curves over time. BI was compared among study groups using Kruskal Wallis test. Confidence interval was established at 95%, thus a p-value less than 0.05 was considered to be statistically significant.

### Ethical considerations

The protocol for this study was reviewed and approved by the Universidade Federal do Rio Grande do Norte Ethical Committee (CAA 06189612.9.0000.5537).

## Results

A total of 543,677 new cases of leprosy were reported in Brazil from 2001–2013, of which 99.5% (n = 541,090) had complete data available and were included in this study. Of those leprosy cases, 54.8% were men, 6.4% were children under 15 years and 17.5% were 60 or more years of age. Most cases (89%) were evaluated for disability grade at diagnosis, of whom 64% (n = 307,834) had no disability and 6% had disability grade 2. NCDR increased from 2001 to 2003, and then progressively decreased to 15.68 new cases/100,000 population in 2013. The decrease in NCDR for both women and men was similar over the 13 years (p = 0.061). The proportion of newly diagnosed leprosy cases who were MB increased 11.6%. The percentage of new cases with 60 or more years of age increased 6% while the proportion of new cases in children under 15 years of age was variable (Table 1).

**Table 1:** Annual indicators for leprosy in Brazil (2001–2013)

Year	Number of new cases	NCDR	Sex ratio	% of MB	% of cases by age group (years)	
					< 15	≥ 60
**2001**	45,859	25.97	1.23	53.5	7.8	16.1
**2002**	49,410	27.58	1.20	53.0	7.8	15.6
**2003**	51,759	28.63	1.18	51.3	8.0	15.2
**2004**	50,461	27.51	1.21	52.3	8.0	16.0
**2005**	49,318	26.58	1.19	53.1	8.1	16.1
**2006**	44,885	23.96	1.20	54.0	7.7	17.0
**2007**	41,586	21.95	1.26	55.7	7.5	18.0
**2008**	39,943	20.86	1.28	57.1	7.5	17.9
**2009**	38,229	19.75	1.25	57.8	7.2	18.8
**2010**	32,540	16.64	1.27	58.9	7.1	19.0
**2011**	34,766	17.61	1.30	61.9	6.9	19.7
**2012**	33,904	17.02	1.34	63.9	6.8	20.9
**2013**	31,017	15.68	1.26	65.1	7.7	22.0

NCDR: New Case Detection Rates per 100,000 residents. Sex ratio: quotient of NCDR in men to NCDR in women. MB: multibacillary cases.

During the study period, the number of cases in males was significantly higher than in females (p<0.0001). The odds of presenting with MB leprosy was twice as high in men as in women (OR = 2.36, CI95% = 2.33–2.38), which was similar for MB leprosy patients detected earlier, with disability grade zero at diagnosis (OR = 2.22; CI95% = 2.19–2.25). Patients aged 60 years old or more had twice the odds of being classified as MB compared with those under 60 years of age (OR = 1.99, CI95% = 1.96–2.02); a similar pattern was observed for MB leprosy patients without disabilities (OR = 1.69; CI95% 1.65–1.72).

**Fig 1:**
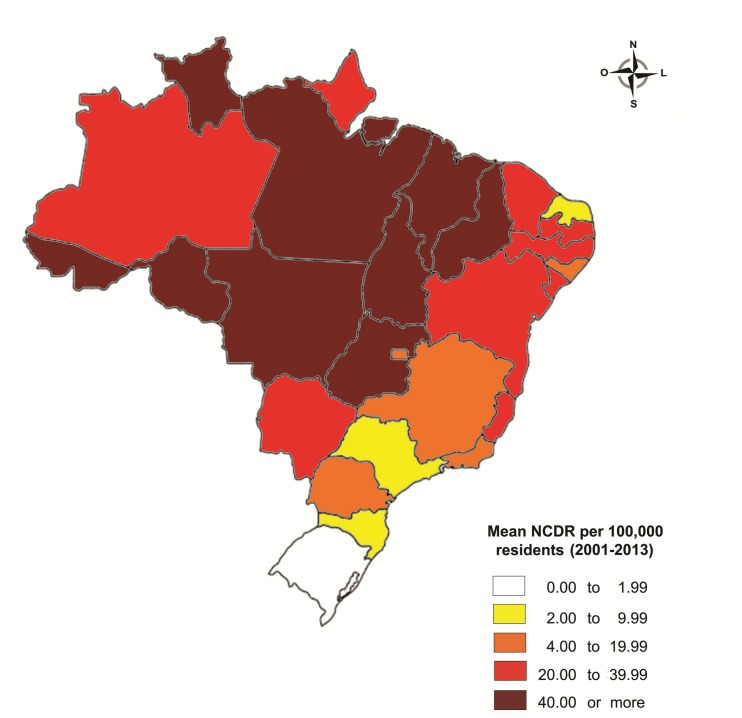
Mean new case detection rates (NCDR) of leprosy by Brazilian state (2001 to 2013).

Mean leprosy NCDRs reached high levels of endemicity in 18 out of 27 Brazilian States, with lower levels observed in the South and Southeast Regions (Fig 1). A significant association between MB cases and age above 59 years was seen in all Brazilian states (S1 Table). This pattern was similar in all states, either with high NCDR such as Mato Grosso (OR = 1.94, CI95% = 1.83–2.07), with a mean NCDR of 104.5 cases/100,000 residents or in states with a low NCDR as Rio Grande do Sul, (OR = 1.86, CI95% = 1.49–2.32), with a mean NCDR of 1.7 cases/100,000 population. When comparing with patients’ sex, the odds of being MB were significantly higher in males than in females regardless of the state’s level of leprosy endemicity. For example, in the North Region, where mean NCDR was 55.6 cases/100,000 population, men had odds of 2.45 (CI95% = 2.39–2.51) higher than women of being MB, whereas in the South Region (mean NCDR = 6.3/100,000 population) the odds were 2.13 (CI95% = 2.01–2.25).

**Fig 2:**
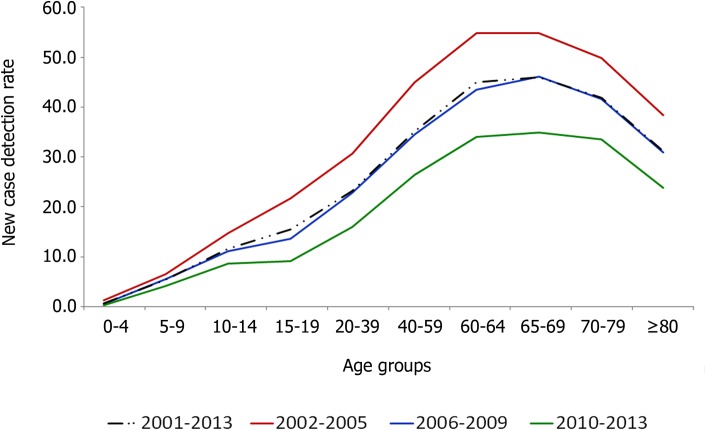
Mean new case detection rates (NCDR) of leprosy by time period, according to age group–Brazil (2001 to 2013).

**Fig 3:**
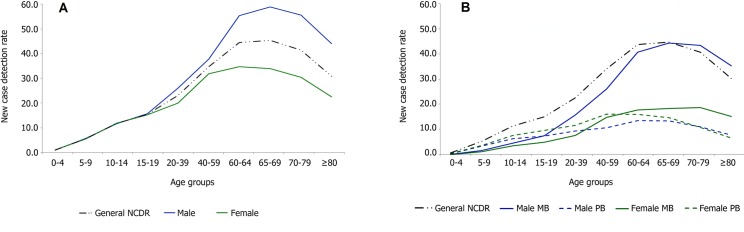
Mean new case detection rates (NCDR) of leprosy in Brazil (2001–2013). 3a) NCDR by sex according to age group. 3b) NCDR by sex and operational classification according to age group. MB: multibacillary, PB: paucibacillary.

Mean NCDRs by age group increased from 1.05 in 0–4 years to a peak of 45.22 cases/100,000 population in 65–69 years (p<0.0001), and was higher for people between 60–69 years of age in the three time periods evaluated (2002–2005, 2006–2009 and 2010–2013). NCDR overall have significantly decreased in all age groups (p<0.0001) (Fig 2). NCDR in both sexes was similar for children and adolescents, but NCDR was significantly higher in adult males than in adult females (Fig 3A), except for those aged 40–59 (p = 0.069). The largest difference in NCDR between males and females was in the 60 years or older age group (p<0.0001).

**Fig 4:**
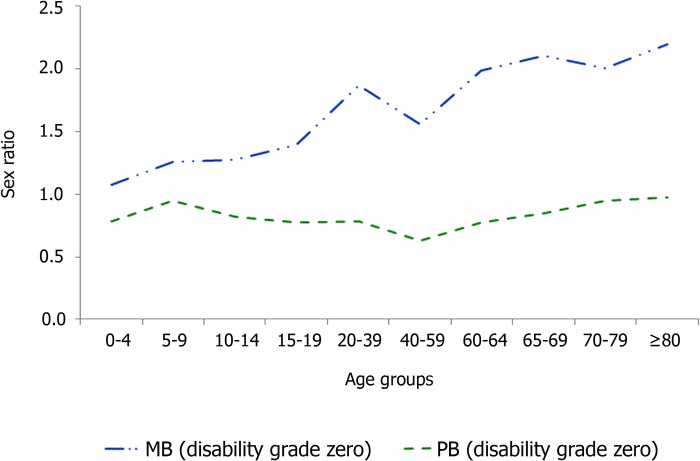
Sex ratio of leprosy cases detected without disabilities, by operational classification according to age group–Brazil. MB: multibacillary, PB: paucibacillary.

Analysis of the mean NCDR (2001–2013) by sex and operational classification according to age group (Fig 3B) indicated that the general mean NCDR was mainly influenced by male MB cases. For females (PB or MB) and for males with PB leprosy, mean NCDR remained under 20 cases/100,000 population in all age groups. However, the NCDR of males with MB, increased steeply above 19 years of age and peaked at 44.8/100,000 population in 65–69 years age group. These differences were also observed for leprosy cases detected with disability grade zero, ie. earlier diagnosed patients, with a progressive increase of NCDR with age for men with MB leprosy, while detection rates remained stable in PB leprosy with predominance of women in all age groups (Fig 4).

**Fig 5:**
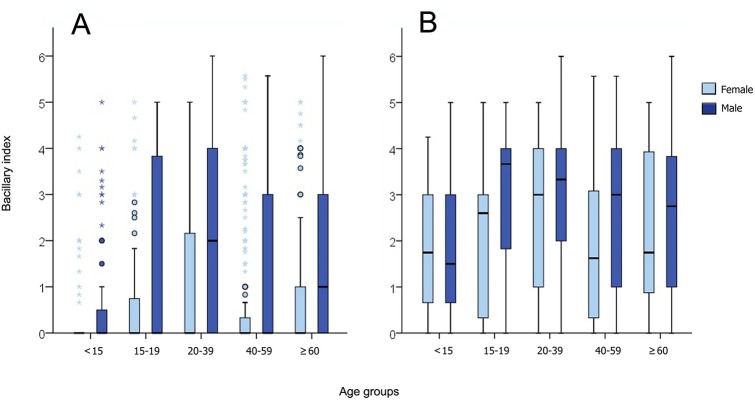
Bacillary index of leprosy cases at diagnosis by sex according to age groups–Ambulatório Souza Araújo, Fiocruz/RJ, Brazil (1990–2014). 5a = All cases; 5b = MB cases. * = extremes, dots = outliers, horizontal bar = median, box = 25–75 percentiles.

Bacillary index (BI) data from 2,253 leprosy cases diagnosed at the ASA/Fiocruz reference center were included (Fig 5A). BI was significantly higher in patients 20–39 years old compared with other age groups (p<0.0001), and it was higher in male than female patients (p<0.0001). Although patients with disability grade 1 or 2 had significantly higher BI than patients with disability grade zero (median test = 90.821, p<0.0001), BI remained significantly higher in males than in females independent of disability grade (median test = 14.423, p<0.0001 for grade zero; median test = 85.383, p<0.0001 for grade 1 or 2). When analyzing only MB cases (Fig 5B), BI was significantly higher in men than women in the 20–39 and 40–59 age groups (p<0.0001).

## Discussion

Decrease in NCDR in countries where leprosy was previously endemic, such as Norway, United States and Japan, was accompanied by an increase in sex ratio and in the percentage of elderly and MB patients among new cases [17,18,19]. In our study, sex ratio was stable over time, but the progressive reduction of annual NCDR together with progressive increase in percentage of elderly and MB cases could indicate decrease in leprosy transmission in Brazil.

Despite the hypothesis that global reduction of leprosy detection rates can reflect a decrease of active case finding activities, an abrupt fall in NCDR was not observed in Brazil as it was in India, where a reduction of 420,000 new cases was observed comparing 2000 to 2006 data, i.e. a reduction of 75% in only six years [4]. In Brazil, the decrease of 20,000 new cases observed between 2003 and 2013 (a 40% reduction), occurred during a period with significant decentralization of leprosy control activities in the country. From 2000 to 2011 it was reported a 284% expansion in the number of health centers that registered patients under treatment (from 3,327 to 9,445) [20].

It is worth noting that from 1980 to 2011 the reduction of leprosy cases in Brazil has occurred as a parabolic curve, without drastic decreases [21]. Moreover, grade 2 disability rate was reduced from 1.40 to 0.99 new leprosy cases per 100,000 population during our study period (2001–2013) [12], suggesting no increase in diagnosis delay. An analysis of leprosy detection rates from 1980 to 2009 in Amazonas State showed that cohorts born in more recent years had smaller risks of leprosy infection than older cohorts, with a declining trend of the relative annual reduction in children that disappeared after 29 years of age [22]. In 2013 and 2014 the Brazilian Ministry of Health introduced specific leprosy detection campaigns for school children from endemic municipalities that involved over than 6 million of students [23], but even though the national NCDR in children was lesser in those years than annual rates observed between 2000 and 2011 [12].

All of these findings may indicate an actual decline in national leprosy incidence, rather than a reduction of new case detection activities. Notwithstanding this decrease in leprosy national NCDR, Brazil is still endemic for leprosy, with hyperendemic or very high NCDR observed in 37% of States in 2015 [24], but with a profile similar to that considered to be of low endemic countries. Therefore, the predominance of multibacillary leprosy in elderly men seems to be a common characteristic to both levels of endemicity.

### Leprosy and sex

Male predominance in leprosy is reported in different parts of the world [1,25–27]. This association could be related to greater exposure of men to the *M*. *leprae* bacillus or by the lack of women undergoing a full physical examination in some cultures [26]. There are also reports of more MB leprosy in men [18,25–27]. In Brazil, this has been documented, and hypothesized to be related to men being less attentive to their health, contributing to later diagnoses with progression to MB leprosy [28]. However, our results show that rates of MB leprosy were higher in men than in women in leprosy *per se* as well as in early diagnosed cases. This association was observed in different states within Brazil regardless of background NCDR of leprosy.

A recent study using random-effects models to evaluate the outcome of infection with ten major human pathogens, including *M*. *leprae*, concluded that differences in response to infection were more associated with physiologic than with behavioral risk factors [29]. Interestingly, in our study sex ratio for leprosy was similar in people less than 20 years of age and then became progressively larger with age, a finding reported by other authors [18,26]. This may be related to a greater exposure of men to the bacillus after childhood, but could be also related to physiologic changes at adolescence, such as estrogen and testosterone levels.

Studies in experimental models of other intracellular pathogens, such as *Leishmania* sp [30], *Paracoccidioides brasiliensis* [31], *Mycobacterium marinum* [32], *M*. *avium* [33] and *M*. *tuberculosis* [34], demonstrated that while estrogen stimulates cellular immune response to these pathogens, with increase in antigen-specific CD4 T cells and IFN-γ secretion, testosterone stimulates production of anti-inflammatory cytokines associated with Th2 response, such as IL-10 and IL-4, and increase in antibody levels. In all experimental models tested more serious lesions and higher parasite burdens were observed in males or in females previously treated with testosterone [30–34].

Our study with data from the ASA/Fiocruz reference center showed that BI was higher in males, even when considering only patients without disability at diagnosis. This suggests that factors other than decreased opportunities for diagnosis may be involved in the development of MB leprosy and higher BI in men. This study found higher BI in men from the age of 20, with significantly higher bacterial load in men 20–59 years of age. A possible explanation for this finding is that testosterone which change during adolescence may be involved in creating an environment that could facilitate *M*. *leprae* growth and yields a higher bacillary load in men. Additional research on sex hormones and leprosy are very important to better discuss these findings.

### Leprosy and age

In Norway, the peak of leprosy NCDR moved from 15–29 years of age in mid 1800’s to people older than 50 years in early 1900s [17], paralleling the overall decrease in the incidence of the disease. At the same time, there was an increase in the percentage of MB cases among the elderly. This is explained by less exposure to *M*. *leprae* among younger people and by a longer incubation period for MB leprosy. Thus, only the previously infected individuals developed especially this type of disease at later ages. This was also observed in other areas where leprosy declined [17–19,27,35], but we found a significant association between MB leprosy and elderly patients in all Brazilian States, including hyperendemic areas from the North and Center-West Regions, indicating that reduced exposure to infection may not be enough to explain this pattern of leprosy.

In addition, it is meaningful to observe that life expectancy in Europe which used to be only 36.3 years in 1850 [36], increased to 50 years of age in Norway at the end of the 1800s [37]. Leprosy incidence peaked in adults in Congo [38] and in children and adolescents in the Philippines [39] when life expectancy in those countries was under 41 years of age [36,40]. Our results in Brazil showed a higher NCDR in elderly in all 3 time periods studied (2002–2005, 2006–2009 and 2010–2013), where life expectancy has been above 70 years of age since the 2000’s [40]. Similar results were observed more recently in Japan [19], Mexico [27] and Korea [41]. These findings may suggest that differences of leprosy NCDR by age could be related to overall life expectancy of the population.

Usually the association between leprosy and the elderly is discussed only as it relates to decreased leprosy transmission and longer incubation period for MB disease, but other potential contributing factors could include age-related immune changes and increased susceptibility to infectious diseases [42]. For example, the elderly can have impaired monocyte and neutrophil function, decrease in CD28 co-stimulatory molecule expression [43], reduction in phagocytic capacity, decreased antigen presentation and a change in cytokine profile from Th1 to Th2 [42]. Thus, key steps required for defense against *M*. *leprae* are potentially compromised with aging, and may be involved in the higher incidence of leprosy in older age groups.

### Integration of active MB case finding in leprosy control programs

As MB patients transmit *M*. *leprae*, we advise targeting specific case detection strategies to men and elderly people to further reduce transmission of leprosy, in addition to preventing disability with early diagnosis and treatment.

In Brazil, the National Health System is based on family health teams, who are responsible for leprosy control among other health issues. These teams are multidisciplinary and usually composed of doctors, nurses and community health workers. The team visits household units for general medical care [44]. This opportunity should be considered an important means to exam elderly people, who sometimes have less access to health services due to decreased mobility and independence [45]. In addition, this knowledge can be useful for active search of the source of infection for leprosy cases under 15 years of age among the elderly men in the households.

Rapid serological tests with high sensitivity to detect MB leprosy are currently available [46, 47]. They are rapid immunochromatographic flow tests that detect IgM antibodies against specific *M*. *leprae* antigens and could be performed as point-of-care serologic evaluations during leprosy active search activities. Although they are not diagnostic tests because they can be positive in healthy people who may never develop leprosy, sera and blood from MB leprosy cases show strongly positive results. Thus, they can be used as an adjunctive tool in diagnostic campaigns, especially for specific population groups where MB leprosy is more frequent.

These strategies can be employed to improve MB leprosy detection and to accelerate the reduction of this important public health problem in Brazil.

### Final comments

This study utilized data generated and entered by hundreds of different people, which needs to be considered in interpretation of the results. An analysis of the leprosy database in Brazil (SINAN) has identified problems linked to updating of data after case notification to the system, such as the date of completion of leprosy therapy; however, no problems where observed related to the software or data transfer [13]. Considering that we used secondary data produced in different settings nationwide, it is important to keep in mind that leprosy classification methodology may vary according to the diagnostic capacity of the center. In primary care settings, diagnosis and operational classification are mainly based on clinical findings, while in references centers, confirmatory and complementary tests, such as skin biopsy and slit-skin smear, can be performed. Nevertheless, during the study period, there were no major changes in the National Program’s recommended diagnosis and classification criteria. Although this may be considered as a limitation, the large number of cases included in the analysis should reduce the impact of differences in classification criteria between centers.

## Supporting information

S1 Table Mean New Case Detection Rates (NCDR) per 100,000 residents (2001–2013) and cumulative number of new cases for the period according to operational classification by Brazilian states and regions. (DOCX)

## References

[1] Britton WJ, Lockwood DNJ. Leprosy. Lancet 2004;363:1209–19. 10.1016/S0140-6736(04)15952-7 15081655

[2] [No authors listed]. World Health Organization (WHO). Global leprosy update, 2015: time for action, accountability and inclusion. Wkly Epidemiol Rec. 2015 9 2;91(35):405–20. 27592500

[3] [No authors listed]. World Health Organization (WHO). Global leprosy update, 2013; reducing disease burden. Wkly Epidemiol Rec. 2014;36 (89):389–400. http://www.ncbi.nlm.nih.gov/pubmed/25202781 25202781

[4] Richardus JH, Habbema JD. The impact of leprosy control on the transmission of M. leprae: is elimination being attained? Lepr Rev. 2007;78:330–337 18309706

[5] Smith WC, van Brakel W, Gillis T, Saunderson P, Richardus JH. The missing millions: a threat to the elimination of leprosy. PLoS Negl Trop Dis. 2015;9(4):e0003658 10.1371/journal.pntd.0003658 25905706 PMC4408099

[6] [No authors listed]. WHO Expert Committee on Leprosy. World Health Organ Tech Rep Ser. 1998;874:1–43 9627517

[7] Brasil. Portal da Saúde. Diretrizes para vigilância, atenção e eliminação da hanseníase como problema de saúde pública. Available at: http://portalsaude.saude.gov.br/images/pdf/2016/fevereiro/04/diretrizes-eliminacao-hanseniase-4fev16-web.pdf (accessed 07 Dec 2016).

[8] Sales AM, Ponce de Leon A, Düppre NC, Hacker MA, Nery JA, Sarno EN et al Leprosy among Patient Contacts: A Multilevel Study of Risk Factors. PLoS Negl Trop Dis. 2011;5(3):e1013 10.1371/journal.pntd.0001013 21423643 PMC3057944

[9] [No authors listed]. Report of the International Leprosy Association Technical Forum. Paris, France, 22–28 February 2002. Int J Lepr Other Mycobact Dis. 2002;70(suppl.):S1–S62. 12125673

[10] Smith CS, Noordeen SK, Richardus JH, Sansarricq H, Cole ST, Soares RC et al A strategy to halt leprosy transmission. Lancet Infect Dis. 2014;14:96–8. 10.1016/S1473-3099(13)70365-7 24457165

[11] Butlin CR, Saunderson P. Children with leprosy. Lepr Rev. 2014;85:69–73. http://www.ncbi.nlm.nih.gov/pubmed/25255609 25255609

[12] Brasil. Portal da Saúde. Situação Epidemiológica—Dados. Indicadores epidemiológicos e operacionais de hanseníase, Brasil 2000–2015. Available from: http://portalarquivos.saude.gov.br/images/pdf/2016/julho/07/Indicadores-epidemiol--gicos-e-operacionais-de-hansen--ase-2000-a-2015.pdf. (accessed 12 Feb 2017).

[13] Galvao PR, Ferreira AT, Maciel MD, De Almeida RP, Hinders D, Schreuder PA et al An evaluation of the Sinan health information system as used by the Hansen's disease control programme, Pernambuco State, Brazil. Lepr Rev. 2008;79:171–182. 18711939

[14] Brasil. Ministério da Saúde. Secretaria de Políticas de Saúde. Departamento de Atenção Básica. Controle da hanseníase na atenção básica: guia prático para profissionais da equipe de saúde da família. 2001. Available at: https://www.yumpu.com/pt/document/view/14734195/controle-da-hanseniase-na-atencao-basica-guia-pratico (accessed 03 Dec 2016).

[15] Leiker DL, McDougall AC. Guia técnico—Baciloscopia da hanseníase. Translation: Ana Tereza Orsi Souza; Martina Ferced Monzon; Sinésio Talhari. 2. ed. Wurzburg. 1987.

[16] Brasil. Instituto Brasileiro de Geografia e Estatística. Projeção da População do Brasil por sexo e idade: 2000–2060. Available at: http://www.ibge.gov.br/home/estatistica/populacao/projecao_da_populacao/2013/default_tab.shtm (accessed 08 Jun 2016).

[17] Irgens LM. Epidemiological aspects and implications of the disappearance of leprosy from Norway; some factors contributing to the decline. Lepr Rev. 1981;52(Suppl 1):147–165. 7040875 10.5935/0305-7518.19810066

[18] Irgens LM, Skjaerven R. Secular trends in age at onset, sex ratio, and type index in leprosy observed during declining incidence rates. Am J Epidemiol. 1985;122:695–705. 3875282 10.1093/oxfordjournals.aje.a114148

[19] Koba A, Ishi N, Mori S, Fine PEl. The decline of leprosy in Japan: patterns and trends 1964–2008. Lepr Rev. 2009;80:432–440. http://www.ncbi.nlm.nih.gov/pubmed/20306642 20306642

[20] Penna ML, Grossi MA, Penna GO. Country profile: leprosy in Brazil. Lepr Rev. 2013;84(4):308–15. https://www.ncbi.nlm.nih.gov/pubmed/?term=PMID%3A+24745130 24745130

[21] Penna ML, de Oliveira ML, Penna GO. The epidemiological behaviour of leprosy in Brazil. Lepr Rev. 2009;80:332–344. https://www.ncbi.nlm.nih.gov/pubmed/19961107 19961107

[22] Penna ML, Pedrosa VL, Dos Santos Pereira E. Leprosy decline in Amazonas State, Brazil. Trop Med Int Health. 2012 2;17(2):244–6. https://www.ncbi.nlm.nih.gov/pubmed/?term=PMID%3A+22035216 10.1111/j.1365-3156.2011.02900.x 22035216

[23] Brasil. Portal da Saúde. Situação Epidemiológica–Dados. Hanseníase, verminoses e tracoma têm cura: a experiência de uma campanha integrada..] Boletim Epidemiológico. 2016; 47(21). Available from: http://portalarquivos.saude.gov.br/images/pdf/2016/maio/12/2015-038---Campanha-publica----o.pdf. (accessed 12 Feb 2017).

[24] Brasil. Portal da Saúde. Situação Epidemiológica—Dados. Taxa de detecção geral de casos novos de hanseníase, estados, Brasil, 2015. Available from: http://portalarquivos.saude.gov.br/images/pdf/2016/julho/07/Taxa-de-detec----o-geral-de-casos-novos-de-hansen--ase--estados--Brasil--2015..pdf. (accessed 12 Feb 2017).

[25] World Health Organization. WHO Expert Committee on Leprosy. World Health Organ Tech Rep Ser. 2012;968:1–61. 22970604

[26] Fine PE. Leprosy: the epidemiology of a slow bacterium. Epidemiol Rev. 1982;4:161–188. 6754406 10.1093/oxfordjournals.epirev.a036245

[27] Larrea MR, Carreño MC, Fine PEM. Patterns and trends of leprosy in Mexico: 1989–2009. Lepr Rev. 2012;83(2):184–194. 22997694

[28] Brasil. Ministério da Saúde. Secretaria de Vigilância em Saúde. Departamento de Vigilância Epidemiológica. Relatório de gestão da Coordenação Geral do Programa Nacional de Controle da Hanseníase–CGPNCH: janeiro 2009 a dezembro de 2010/ Hansen’s Disease Control National Program: Management Report: from January 2009 until December 2010. Brasília–Ministério da Saúde 2011:21–31.

[29] Guerra-Silveira F, Abad-Franch F. Sex bias in infectious disease epidemiology: patterns and processes. PLoS One. 2013;8(4):e62390 10.1371/journal.pone.0062390 23638062 PMC3634762

[30] Snider H, Lezama-Davila C, Alexander J, Satoskar AR. Sex hormones and modulation of immunity against leishmaniasis. Neuroimmunomodulation. 2009;16:106–13. Epub 2009 Feb 11. 10.1159/000180265 19212130 PMC2760305

[31] Pinzan CF, Ruas LP, Casabona-Fortunato AS, Carvalho FC, Roque-Barreira MC. Immunological basis for the gender differences in murine *Paracoccidioides brasiliensis* infection. PLoS One. 2010;5:e10757 10.1371/journal.pone.0010757 20505765 PMC2873977

[32] Yamamoto Y, Saito H, Setogawa T, Tomioka H. Sex differences in host resistance to *Mycobacterium marinum* infection in mice. Infect Immun. 1991;59:4089–4096. 1937768 10.1128/iai.59.11.4089-4096.1991 PMC259001

[33] Yamamoto Y, Tomioka H, Sato K Saito H, Yamada Y, Setogawa T. Sex differences in the susceptibility of mice to infection induced by *Mycobacterium intracellulare*. Am Rev Respir Dis. 1990;142:430–433. 10.1164/ajrccm/142.2.430 2382907

[34] Bini EI, Mata Espinosa D, Marquina Castillo B, Barrios Payán J, Colucci D, Cruz AF et al. The Influence of Sex Steroid Hormones in the Immunopathology of Experimental Pulmonary Tuberculosis. PLoS One. 2014;9(4):e93831 eCollection 2014. http://www.ncbi.nlm.nih.gov/pubmed/24722144 10.1371/journal.pone.0093831 24722144 PMC3983091

[35] Irgens LM, Melo Caeiro F, Lechat MF. Leprosy in Portugal 1946–80: epidemiologic patterns observed during declining incidence rates. Lepr Rev. 1990;61:32–49. 2319900 10.5935/0305-7518.19900005

[36] Riley JC. Estimates of Regional and Global Life Expectancy, 1800–2001. Popul Dev Rev. 2005;31(3):537–543.

[37] Norwegian Institute of Public Health. Life expectancy in Norway–fact sheet. Disponible at https://www.fhi.no/en/hn/cause-of-death-and-life-expectancy/life-expectancy-in-norway—fact-sh/ (accessed 20 jun 2016)

[38] Browne SG. The age of onset of leprosy. Int J Lepr. 1965;33(3):267–72. http://www.ncbi.nlm.nih.gov/pubmed/5854821 5854821

[39] Guinto RS, Rodriguez JN, Doull JA, De Gula L. The trend of leprosy in Cordova and Talisay, Cebu Province, Philipines. Int J Lepr. 1954;22(4):409–30. http://www.ncbi.nlm.nih.gov/pubmed/14366787 14366787

[40] The World Bank. World Development Indicators. Life expectancy at birth, total (years). Disponible at http://data.worldbank.org/indicator/SP.DYN.LE00.IN (accessed 24 Apr 2016)

[41] Lee J, Kim JP, Nishikiori N, Fine PEM. The decline of leprosy in the Republic of Korea; patterns and trends 1977–2013. Lepr Rev. 2015;86(4):316–327. http://www.ncbi.nlm.nih.gov/pubmed/26964427 26964427

[42] Heppner HJ, Cornel S, Peter W, Bahrmann P, Singler K. Infections in the elderly. Crit Care Clin 2013;29:757–74. http://www.tandfonline.com/servlet/linkout?suffix=cit0002&dbid=8&doi=10.1080%2F21505594.2016.1140296&key=23830661 10.1016/j.ccc.2013.03.016 23830661

[43] Chou JP, Effros RB. T cell replicative senescence in human aging. Curr Pharm Des. 2013;19(9):1680–1698. 23061726 10.2174/138161213805219711 PMC3749774

[44] James M, Harris MJ. Brazil's family health strategy—delivering community-based primary care in a universal health system. N Engl J Med. 2015;372:2177–2181. 10.1056/NEJMp1501140 26039598

[45] Mendes ACG, Sá DAD, Miranda GMD, Lyra TM e Tavares RA. Assistência pública de saúde no contexto da transição demográfica brasileira: exigências atuais e futuras/ The public healthcare system in the context of Brazil's demographic transition: current and future demands. Cad Saúde Pública. 2012;28(5):955–64. http://www.ncbi.nlm.nih.gov/pubmed/22641518 22641518 10.1590/s0102-311x2012000500014

[46] Buhrer-Sekula S, Smits HL, Gussenhoven GC et al Simple and fast lateral flow test for classification of leprosy patients and identification of contacts with high risk of developing leprosy. Clin Microbiol. 2003;41:1991–1995 https://www.ncbi.nlm.nih.gov/pmc/articles/PMC154748/10.1128/JCM.41.5.1991-1995.2003 PMC154748 12734239

[47] Cardoso LP, Dias RF, Freitas AA, Hungria EM, Oliveira RM, Collovati M et al Development of a quantitative rapid diagnostic test for multibacillary leprosy using smart phone technology. BMC Infect Dis. 2013;13:497 https://www.ncbi.nlm.nih.gov/pubmed/?term=PMID%3A+24152601 10.1186/1471-2334-13-497 24152601 PMC3870957

