# Supplementary material for: Multibacillary leprosy by population groups in Brazil: Lessons from an observational study
Source: PLoS Negl Trop Dis. 2017 Feb 13;11(2):e0005364. doi: 10.1371/journal.pntd.0005364 (PMC5325588; doi:10.1371/journal.pntd.0005364)
Supplement: S1 Table — (DOCX) [file pntd.0005364.s001.docx]

**S1 Table. Mean New Case Detection Rates (NCDR) per 100,000 residents (2001-2013) and cumulative number of new cases for the period according to operational classification by Brazilian states and regions.**

| **State** | **Mean NCDR** | **Number of new cases (2001-2013)** | | | | **OR** | **95%CI** | **Number of new cases (2001-2013)** | | | | **OR** | **95%CI** |
| --- | --- | --- | --- | --- | --- | --- | --- | --- | --- | --- | --- | --- | --- |
|  |  | **Male** | | **Female** | |  |  | **≥ 60** | | **< 60** | |  |  |
|  |  | **MB** | **PB** | **MB** | **PB** |  |  | **MB** | **PB** | **MB** | **PB** |  |  |
| Rondônia | 69.1 | 4,881 | 3,274 | 2,644 | 3,574 | **2.01** | 1.88 - 2.15 | 1,115 | 439 | 6,237 | 6,495 | **2.64** | 2.35 - 2.97 |
| Acre | 43.7 | 1,673 | 773 | 679 | 876 | **2.79** | 2.44 - 3.18 | 277 | 88 | 2,071 | 1,561 | **2.37** | 1.85 - 3.04 |
| Amazonas | 25.9 | 4,328 | 2,708 | 1,595 | 2,962 | **2.96** | 2.74 - 3.20 | 815 | 406 | 4,935 | 5,239 | **2.13** | 1.88 - 2.41 |
| Roraima | 52.5 | 1,157 | 569 | 460 | 569 | **2.51** | 2.14 - 2.94 | 249 | 79 | 1,392 | 1,115 | **2.52** | 1.93 - 3.29 |
| Para | 65.1 | 24,228 | 12,582 | 10,686 | 13,939 | **2.51** | 2.40 - 2.59 | 5,303 | 1,922 | 29,132 | 24,986 | **2.36** | 2.24 - 2.50 |
| Amapá | 25.2 | 915 | 483 | 345 | 459 | **2.52** | 2.11 - 3.01 | 169 | 79 | 1,014 | 825 | **1.74** | 1.31 - 2.30 |
| Tocantins | 84.8 | 4,765 | 3,856 | 2,282 | 4,172 | **2.25** | 2.11 - 2.41 | 1,460 | 859 | 5,362 | 7,295 | **2.31** | 2.11 - 2.53 |
| **North Region** | 55.6 | **41,947** | **24,245** | **18,691** | **26,551** | **2.45** | **2.39 - 2.51** | **9,388** | **3,872** | **50,143** | **47,516** | **2.29** | **2.20 - 2.39** |
| Maranhão | 71.9 | 22,560 | 10,390 | 12,187 | 13,176 | **2.34** | 2.26 - 2.42 | 7,384 | 2,553 | 27,235 | 21,422 | **2.27** | 2.16 - 2.38 |
| Piauí | 48.2 | 5,733 | 4,359 | 3,348 | 6,203 | **2.43** | 2.3 - 2.58 | 2,429 | 1,530 | 6,434 | 8,806 | **2.17** | 2.02 - 2.33 |
| Ceara | 28.9 | 11,606 | 4,743 | 6,943 | 7,850 | **2.76** | 2.64 - 2.89 | 5,419 | 2,103 | 13,177 | 10,659 | **2.08** | 1.90 - 2.20 |
| Rio Grande do Norte | 9.8 | 1,159 | 659 | 778 | 1,248 | **2.82** | 2.47 - 3.21 | 582 | 292 | 1,394 | 1,669 | **2.38** | 2.03 - 2.79 |
| Paraíba | 22.1 | 3,055 | 2,113 | 1,868 | 3,471 | **2.68** | 2.48 - 2.90 | 1,437 | 800 | 3,450 | 4,803 | **2.50** | 2.27 - 2.75 |
| Pernambuco | 35.4 | 11,577 | 7,592 | 7,600 | 12,772 | **2.56** | 2.46 - 2.66 | 4,121 | 2,535 | 14,934 | 17,918 | **1.95** | 1.84 - 2.05 |
| Alagoas | 13.5 | 1,563 | 1,008 | 988 | 1,810 | **2.84** | 2.54 - 3.17 | 541 | 304 | 2,008 | 2,548 | **2.25** | 1.93 - 2.62 |
| Sergipe | 23.4 | 1,808 | 1,256 | 1,022 | 2,083 | **2.93** | 2.64 - 3.25 | 664 | 426 | 2,112 | 2,984 | **2.20** | 1.92 - 2.51 |
| Bahia | 20.4 | 11,213 | 7,334 | 7,376 | 11,051 | **2.29** | 2.19 - 2.38 | 4,105 | 2,354 | 14,541 | 16,305 | **1.95** | 1.85 - 2.06 |
| **Northeast Region** | **31.2** | **70,274** | **39,454** | **42,110** | **59,664** | **2.52** | **2.48 - 2.56** | **26,682** | **12,897** | **85,285** | **87,114** | **2.11** | **2.06 - 2.16** |
| Minas Gerais | 11.8 | 12,717 | 3,808 | 8,363 | 5,561 | **2.22** | 2.11 - 2.33 | 5,311 | 1,461 | 15,489 | 7,962 | **1.86** | 1.75 - 1.99 |
| Espirito Santo | 35.5 | 4,401 | 4,105 | 2,269 | 5,418 | **2.56** | 2.39 - 2.73 | 1,644 | 1,207 | 4,941 | 8,443 | **2.32** | 2.14 - 2.52 |
| Rio de Janeiro | 14.9 | 10,134 | 5,496 | 6,066 | 8,861 | **2.69** | 2.57 - 2.82 | 3,943 | 2,624 | 12,077 | 11,848 | **1.47** | 1.39 - 1.55 |
| São Paulo | 5.5 | 10,671 | 6,137 | 5,275 | 7,540 | **2.48** | 2.37 - 2.60 | 4,251 | 2,434 | 11,399 | 11,481 | **1.75** | 1.66 - 1.86 |
| **Southeast Region** | **10.2** | **37,923** | **19,546** | **21,973** | **27,380** | **2.41** | **2.35 - 2.47** | **15,149** | **7,726** | **43,906** | **39,734** | **1.77** | **1.72 - 1.83** |
| Paraná | 12.7 | 7,184 | 2,509 | 4,360 | 3,315 | **2.17** | 2.04 - 2.32 | 3,297 | 1,092 | 8,227 | 4,767 | **1.74** | 1.62 - 1.89 |
| Santa Catarina | 3.4 | 1,179 | 377 | 671 | 489 | **2.27** | 1.93 - 2.68 | 450 | 159 | 1,377 | 714 | **1.46** | 1.19 - 1.79 |
| Rio Grande do Sul | 1.7 | 989 | 240 | 830 | 371 | **1.84** | 1.52 - 2.22 | 588 | 123 | 1,254 | 489 | **1.86** | 1.49 - 2.32 |
| **South Region** | **6.3** | **9,352** | **3,126** | **5,861** | **4,175** | **2.13** | **2.01 - 2.25** | **4,335** | **1,374** | **10,858** | **5,970** | **1.73** | **1.62 - 1.85** |
| Mato Grosso do Sul | 28.6 | 3,164 | 1,639 | 1,900 | 1,849 | **1.87** | 1.72 - 2.05 | 1,302 | 598 | 3,799 | 2,965 | **1.69** | 1.52 - 1.89 |
| Mato Grosso | 104.5 | 13,809 | 8,923 | 7,360 | 9,261 | **1.94** | 1.87 - 2.02 | 3,634 | 1,799 | 17,358 | 16,744 | **1.94** | 1.83 - 2.07 |
| Goiás | 47.7 | 16,048 | 4,675 | 9,956 | 5,696 | **1.96** | 1.87 - 2.05 | 4,745 | 1,157 | 20,856 | 9,424 | **1.85** | 1.73 - 1.98 |
| Distrito Federal | 10.6 | 1,428 | 626 | 883 | 823 | **2.12** | 1.86 - 2.43 | 301 | 106 | 1,731 | 1,229 | **2.01** | 1.59 - 2.54 |
| **Center-West Region** | **49.9** | **34,449** | **15,863** | **20,099** | **17,629** | **1.90** | **1.85 - 1.95** | **9,982** | **3,660** | **43,744** | **30,362** | **1.89** | **1.81 - 1.97** |
| **Brazil** | **21.9** | **193,945** | **102,234** | **108,734** | **135,399** | **2.36** | **2.33 - 2.38** | **65,536** | **29,529** | **233,936** | **210,696** | **1.99** | **1.96 - 2.02** |

The odds of being multibacillary are significantly higher for males as compared to females and for patients aged ≥ 60 years as compared to patients under 60 years, in all regions. MB: multibacillary, PB: paucibacillary. OR: odds ratio. CI: Confidence interval.

.
